# Helping women to good health: breast cancer, omega-3/omega-6 lipids, and related lifestyle factors

**DOI:** 10.1186/1741-7015-12-54

**Published:** 2014-03-27

**Authors:** Michel de Lorgeril, Patricia Salen

**Affiliations:** 1 Laboratoire TIMC-IMAG, CNRS UMR 5525, PRETA Cœur and Nutrition, and Faculté de Médecine, Université Joseph Fourier, Grenoble, France

**Keywords:** Polyphenols, Organic foods, Endocrine disruptors, Insulin, Diabetes, Cholesterol, Hypertension, Mediterranean diet

## Abstract

In addition to genetic predisposition and sex hormone exposure, physical activity and a healthy diet play important roles in breast cancer (BC). Increased intake of omega-3 fatty acids (n-3) associated with decreased omega-6 (n-6), resulting in a higher n-3/n-6 ratio compared with the western diet, are inversely associated with BC risk, as shown by Yang *et al*. in their meta-analysis in *BMC Cancer*. High consumption of polyphenols and organic foods increase the n-3/n-6 ratio, and in turn may decrease BC risk. Intake of high fiber foods and foods with low glycemic index decreases insulin resistance and diabetes risk, and in turn may decrease BC risk. The modernized Mediterranean diet is an effective strategy for combining these recommendations, and this dietary pattern reduces overall cancer risk and specifically BC risk. High-risk women should also eliminate environmental endocrine disruptors, including those from foods. Drugs that decrease the n-3/n-6 ratio or that are suspected of increasing BC or diabetes risk should be used with great caution by high-risk women and women wishing to decrease their BC risk.

Please see related article: http://www.biomedcentral.com/1471-2407/14/105/abstract.

## Introduction

Breast cancer (BC) remains one of the commonest cancers – one in eight women will be diagnosed with BC in her lifetime [1] – and a leading cause of death from cancer. However, it remains a significant scientific and medical challenge. One of the crucial gaps identified is how to implement a sustainable preventive lifestyle strategy [2]. Both risk factors and protective factors must be considered. Some risk factors, such as genetic predisposition, cannot be modified, whereas others (unhealthy diet, sedentary lifestyle) can be avoided. Increasing protective factors may be crucial for women at high risk (as evaluated by the National Cancer Institute’s (NCI’s) BC Risk Assessment Tool [3]) and in preventing recurrence and improving survival after BC diagnosis. Decreasing the length of time a woman’s breast tissue is exposed to estrogens may help prevent BC [1-3], although the main ways of achieving this (first pregnancy before the age of 20 years, breast feeding, late menstruation and early menopause) are difficult to control.

There are strong links between environmental/lifestyle factors and BC, suggesting that modifying these factors may result in decreasing BC risk, although there has been no randomized trial that clearly demonstrated this. As changing these factors has been shown also to definitely reduce the risk of fatal diseases, notably cardiovascular diseases in randomized trials, it is reasonable to propose these changes to high-risk women and to women who wish to reduce their BC risk.

For instance, dietary fats have been extensively studied in the prevention of BC [4]. Neither animal fat nor a low-fat diet has been linked to BC risk, whereas marine omega-3 fatty acids (n-3) may be protective [4]. In a meta-analysis of 21 independent prospective cohort studies, Zheng *et al*. found a significant reduction of BC risk with marine n-3 [5]. However, this meta-analysis highlights the difficulties in assessing the effects of specific dietary fats on BC risk. In subgroup analyses, Zheng *et al*. found that the inverse associations between marine n-3 and BC risk were significant only in post-menopausal women, were stronger in East Asian populations compared with western populations and were more evident without adjustment for body mass index (BMI). This suggests that marine n-3 may influence BC risk partly through an effect on BMI or related factors (insulin or adipokines), whereas the importance of BMI on BC risk during the pre-menopausal and post-menopausal periods is still a matter of controversy [6]. The East Asian population issue suggests that other foods/nutrients and related factors may be involved. For instance, omega-6 fatty acids (n-6) may play a role in BC risk [7]. It has long been suspected that n-6 increase the risk of cancers, and this has been confirmed in controlled trials in which n-6 intakes were modified. In the Los Angeles Trial, there were more cancers in the experimental group with high n-6 intake, whereas in the Lyon Diet Heart Study, there were fewer cancers in the group with low n-6 [8,9]. Thus, when analyzing the associations between n-3 and BC risk, it is crucial that n-6 is included in the analyses, as Yang *et al*. did in their recent study published in *BMC Cancer*[10].

### Breast cancer risk and n-3/n-6 ratios

Yang *et al*. used the ratio of n-3/n-6 in a meta-analysis comprising 274,135 women with a total of 8,331 BC events from 11 independent prospective studies [10]. Women with a higher n-3/n-6 ratio had a significantly lower risk of BC: pooled RR 0.90, 95% CI 0.82 to 0.99. When the authors analyzed only dietary intake, they found a 6% reduction in BC risk per one-tenth increment of the n-3/n-6 ratio. The association between the blood phospholipid n-3/n-6 ratio and BC risk (in four studies only) did not reach statistical significance. The lack of a significant relationship between BC risk and blood n-3/n-6 ratio is not unexpected, because n-3 and n-6 measured in phospholipids do not accurately reflect dietary intake. Several factors (discussed in the next paragraph) interfere with the levels of both n-3 and n-6 in each class of phospholipids. In addition, the fatty acid composition of each phospholipid is not identical: it differs in serum, red cell membranes, and mitochondria, for example, and these various phospholipids have a different physiology and a different effect (if any) on cancers [7]. There are nonetheless correlations between dietary n-6 and n-3 and the corresponding fatty acids in blood and cells, and it is not illogical to pool data from diet and blood when analyzing relationships with BC risk. It is, however, crucial to bear these differences in mind when interpreting the results, and potential confounders should be included in the analyses if possible; that is, if they have been recorded in a timely and accurate fashion. In the next section, we highlight some lifestyle, environmental, and pharmacological factors that influence n-3/n-6 ratios, and examine their relationships with BC risk. In most studies included in the meta-analysis by Yang *et al*., these factors were not measured, which is likely to have weakened the associations between n-3/n-6 ratio and BC risk.

### Factors influencing n-3/n-6 ratios and BC risk

Among the main risk factors of BC, estrogen exposure, lack of physical exercise and being overweight are well known [1-3].

Studies support an association between endogenous sex hormone levels and BC risk for post-menopausal women, whereas the association is less clear for pre-menopausal women [11]. One possible explanation is that the high estrogen levels present before menopause increase blood marine n-3 [12], which in turn may partly counteract the effect of estrogens, as marine n-3 are protective [5,7,10].

Besides increased intake of n-3 and decreased intake of n-6 through consumption of foods rich in n-3 and poor in n-6 [7-9], other substances are known to affect the n-3/n-6 ratio. The endogenous synthesis of marine n-3 from their plant substrate alpha-linolenic acid (ALA) is stimulated by the plant pigment polyphenols found in purple fruits such as grapes (and wines), plums, and blueberries [13-15]. Polyphenol flavonoids increase marine n-3 by 30% without altering n-6 levels, resulting in a significant increase in the n-3/n-6 ratio. As expected from the above data, flavonoids are associated with a decreased BC risk [16]. These data are encouraging, as it is difficult to accurately determine the intake of each flavonoid, and this probably weakens the association between specific flavonoids and BC risk. In addition, the bioavailability and biological effect of flavonoids are dependent on many factors, including the gut microbiota [17], which again is likely to weaken the associations. A typical example of such complexity is provided by the soy flavonoids.

Soy isoflavones, a major class of phytoestrogens, may reduce BC risk, but epidemiologic studies have yielded inconsistent results [18]. A meta-analysis suggests that protection is only observed in studies conducted in East Asian and not in western populations [18], suggesting that protection may require that women consume the high levels of soy typical of East Asian diets. In addition, the food source of isoflavones, timing of exposure to isoflavones (starting at or not before adolescence), the woman’s menopausal status, and the ability of the gut microbiota to transform isoflavones into equol probably modify the association between soy isoflavones and BC risk. Equol is more biologically active (that is, more anti-estrogenic) than dietary isoflavones, but only about 30% of women in the USA and Australia are equol producers [19]. Finally, the small studies examining whether equol exposure is associated with BC risk have produced inconsistent results [20]. Other phytoestrogens more characteristic of the western diet are the polyphenol lignans present in seeds, grains, fruits, and vegetables. High dietary lignans or high exposure to enterolignans, the metabolites resulting from transformation by the gut microbiota, are associated with lower BC risk and better survival in post-menopausal BC [21,22]. This is not unexpected, as we have shown in rats that lignans increase blood n-3 without affecting n-6, resulting in a higher n-3/n-6 ratio [7,13].

Another crucial point is the fact that organic plant foods contain more polyphenols than similar conventional foods [23-26]. Organic animal fat also - for instance, milk and milk products - do have a higher n-3/n-6 ratio than conventional products [27-29]. Organic-fed animals mainly consume non-contaminated fresh grass with a high polyphenol content, rather than polyphenol-poor concentrates potentially contaminated with pesticides, and this may partly explain the favorable n-3/n-6 ratio [30,31].

Regarding food contaminants, recent studies have shown a strong association between estrogenic PCBs (polychlorobiphenyls) congeners and BC risk [32]. In this context, it is crucial to identify ’hidden’ groups of women whose occupational exposure to carcinogens is underrepresented in epidemiological studies. Studies have recently shown significant associations between BC risk and endocrine disruptors in women with specific professional exposure [33]. Endocrine disruptors (particularly phthalates) increase insulin resistance, and the risk of diabetes and obesity [34], all of which increase BC risk [1-3].

Other commonly encountered substances that potentially influence both n-3/n-6 ratio and BC risk are the cholesterol-lowering statins. Statins decrease n-3/n-6 ratio [35], increase insulin resistance and diabetes risk [36], lower cholesterol, and are toxic to mitochondria [37]. Whether or not statins, by modifications of the above processes [35-38], are involved in increased BC risk, remains an area of controversy. Therefore, when weighing up the benefits and potential risks of statins, caution should be applied prior to prescription. Following the same line of reasoning, high-risk women should do everything they can to reduce their risk of insulin resistance, metabolic syndrome and diabetes [1-3]. Beside optimal physical activity, which is a well-known strategy for decreasing both diabetes and BC [1-3] risks, high fiber intake, high flavonoids, and high n-3 are all inversely associated with diabetes risk. Accordingly, fiber intake, flavonoids [16], and n-3 [5,7,10] are inversely associated with BC risk. Finally, consumption of foods with a lower glycemic impact – that is, a low glycemic index (GI) – is associated with a lower incidence of diabetes and a lower BC risk [1-3].

These data are highly consistent, and the combination of high fiber, high n-3/n-6 ratio, high polyphenols, and low-GI foods represents what many experts call a healthy dietary pattern – for instance, the Mediterranean diet – which has been associated with a lower BC risk and better survival for women with early-stage BC.

### Reducing BC risk

In summary (see Table 1), the accumulated evidence shows that in addition to genetic predisposition and estrogen exposure, a number of lifestyle, environmental, and pharmacological factors play an important role in BC risk and BC survival. Optimal physical activity decreases insulin resistance, diabetes risk, and the risk and progression of BC [39]. Adhering to a healthy dietary pattern – a modernized Mediterranean diet [40], or a similar healthy diet adapted to specific populations, such as the Okinawan diet in East Asians [41] – has been shown to be effective. Emphasis should be given to increasing plant and animal (including marine) n-3, and decreasing plant and animal n-6. High polyphenol consumption, in particular of flavonoids, which increase the synthesis of marine n-3 and result in a higher n-3/n-6 ratio, is associated with lower BC risk. To reduce insulin resistance and diabetes, which are associated with an increased BC risk, women should increase their consumption of n-3 and fiber and favor low-GI foods. In addition, organic foods have been shown to contain more polyphenols and have a higher n-3/n-6 ratio than non-organic foods. As a higher n-3/n-6 ratio has been associated with decreased BC risk, consumption of organic foods may be beneficial. Care must be taken to reduce (professional and non-professional) exposure to environmental chemical contaminants. Drugs that decrease the n-3/n-6 ratio, and/or increase insulin resistance and diabetic risk (in particular statins) should be taken with caution.

**Table 1:** How to reduce breast cancer risk and improve survival after diagnosis

**Item**	**Recommendations**
Exposure to sex steroids	Decrease
Physical activity	Adopt optimal level^1^
Modernized Mediterranean diet^2^	Full adherence
Plant and marine n-3	Increase^3^
Plant and animal n-6	Decrease^3^
Polyphenols	Increase^3^
Fiber	Increase^3^
High GI foods	Decrease^3^
Alcohol (no more than 1 drink/day)	Preferably wine
Organic foods (plant and animal sources)	May be beneficial
Exposure to endocrine disruptors	Decrease where possible
Insulin resistance and diabetic risk	Decrease^4^
Age-associated weight gain	Limit^4^
Drugs	
Cholesterol-lowering statins	Use caution
Drugs suspected to increased BC risk	Use caution
Drugs suspected to increased insulin resistance and diabetic risk	Use caution

BC, breast cancer; GI, glycemic index.

^1^Tailored to the capacity of each individual.

^2^Consider an alternative healthy diet in specific populations; Okinawan diet for East Asians for instance.

^3^Compared with a western-style diet.

^4^By adhering to the modernized Mediterranean diet (see text).

## Conclusions

National and international cancer associations regularly release anti-cancer guidelines. For instance, every five years the American Cancer Society publishes its Nutrition and Physical Activity Guidelines, which reflect current scientific evidence and focus on recommendations for individual choices regarding diet and physical activity patterns [42]. These Guidelines state: ‘*for people who do not use tobacco, the most important modifiable determinants of cancer risk are weight control, dietary choices, and levels of physical activity*’ and ‘*although genetic susceptibility influences the risk of cancer, most of the variation in cancer risk across populations and among individuals is due to factors that are not inherited*’. Regarding BC specifically, the Guidelines state, ‘*the best advice to reduce the risk of breast cancer is to engage in regular, intentional physical activity; to minimize lifetime weight gain through the combination of caloric restriction (in part by consuming a diet rich in vegetables and fruits) and regular physical activity; and to avoid or limit intake of alcoholic beverages’*[42].

We agree wholeheartedly with this advice. However, we feel it is time to go further and to be more specific. A specific healthy dietary pattern such as the modernized Mediterranean diet, and not simply “*consuming a diet rich in vegetables and fruits*”, should be adopted to decrease BC risk. This is also an effective way of maintaining a healthy weight, and preventing diabetes and cardiovascular disease. The focus must be on the n-3/n-6 ratio and polyphenols. Organic foods are shown to contain higher levels of these than non-organic foods, and so may be beneficial. Great care should be taken when using drugs that potentially increase BC risk. This also applies to BC survivors to prevent recurrence and improve survival [43].

## Abbreviations

ALA: Alpha-linolenic acid; BC: Breast cancer; GI: Glycemic index; n-3: Omega-3 fatty acids; n-6: Omega-6 fatty acids.

## Competing interests

The authors declare that they have no competing interests.

## Authors’ contributions

MdeL drafted the manuscript. PS critically revised the manuscript, and gave final approval for publication. Both authors read and approved the final manuscript.

## References

[B1] Matsen CB Neumayer LA Breast cancer: a review for the general surgeon JAMA Surg 2013 148 971 979 10.1001/jamasurg.2013.3393 23986370

[B2] Eccles SA Aboagye EO Ali S Anderson AS Armes J Berditchevski F Blaydes JP Brennan K Brown NJ Bryant HE Bundred NJ Burchell JM Campbell AM Carroll JS Clarke RB Coles CE Cook GJ Cox A Curtin NJ Dekker LV Silva Idos S Duffy SW Easton DF Eccles DM Edwards DR Edwards J Evans D Fenlon DF Flanagan JM Foster C Critical research gaps and translational priorities for the successful prevention and treatment of breast cancer Breast Cancer Res 2013 15 R92 10.1186/bcr3493 24286369 PMC3907091

[B3] Breast Cancer Risk Assessment Tool - National Cancer Institutehttp://www.cancer.gov/bcrisktool/ (Accessed March 11, 2014)

[B4] Khaw KT Dietary fats and breast cancer risk BMJ 2013 347 f4518 10.1136/bmj.f4518 23861431

[B5] Zheng JS Hu XJ Zhao YM Yang J Li D Intake of fish and marine n-3 polyunsaturated fatty acids and risk of breast cancer: meta-analysis of data from 21 independent prospective cohort studies BMJ 2013 346 f3706 10.1136/bmj.f3706 23814120

[B6] Cheraghi Z Poorolajal J Hashem T Esmailnasab N Doosti Irani A Effect of body mass index on breast cancer during premenopausal and postmenopausal periods: a meta-analysis PLoS One 2012 7 e51446 10.1371/journal.pone.0051446 23236502 PMC3517558

[B7] de Lorgeril M Salen P New insights into the health effects of dietary saturated and omega-6 and omega-3 polyunsaturated fatty acids BMC Med 2012 10 50 10.1186/1741-7015-10-50 22613931 PMC3394202

[B8] Pearce ML Dayton S Incidence of cancer in men on a diet high in polyunsaturated fat Lancet 1971 1 464 467 4100347 10.1016/s0140-6736(71)91086-5

[B9] de Lorgeril M Salen P Martin JL Monjaud I Boucher P Mamelle N Mediterranean dietary pattern in a randomized trial: prolonged survival and possible reduced cancer rate Arch Intern Med 1998 158 1181 1187 10.1001/archinte.158.11.1181 9625397

[B10] Yang B Ren XL Fu YQ Gao JL Duo L Ratio of n-3/n-6 PUFAs and risk of breast cancer: a meta-analysis of 274,135 adult females from 11 independent prospective studies BMC Cancer 2014 14 105 10.1186/1471-2407-14-105 24548731 PMC4016587

[B11] Kaaks R Tikk K Sookthai D Schock H Johnson T Tjønneland A Olsen A Overvad K Clavel-Chapelon F Dossus L Baglietto L Rinaldi S Chajes V Romieu I Boeing H Schütze M Trichopoulou A Lagiou P Trichopoulos D Palli D Sieri S Tumino R Ricceri F Mattiello A Buckland G Ramón Quirós J Sánchez MJ Amiano P Chirlaque MD Premenopausal serum sex hormone levels in relation to breast cancer risk, overall and by hormone receptor status-results from the EPIC cohort Int J Cancer 2013 10.1002/ijc.28528 24155248

[B12] Giltay EJ Gooren LJ Toorians AW Katan MB Zock P Docosahexaenoic acid concentrations are higher in women than in men because of estrogenic effects Am J Clin Nutr 2004 80 1167 1174 15531662 10.1093/ajcn/80.5.1167

[B13] Toufektsian MC Salen P Laporte F Tonelli C de Lorgeril M Dietary flavonoids increase plasma very long-chain (n-3) fatty acids in rats J Nutr 2011 141 37 41 10.3945/jn.110.127225 21068183

[B14] di Giuseppe R de Lorgeril M Salen P Laporte F Di Castelnuovo A Krogh V Siani A Arnout J Cappuccio FP van Dongen M Donati MB de Gaetano G Iacoviello L European Collaborative Group of the IMMIDIET Project Alcohol consumption and n-3 polyunsaturated fatty acids in healthy men and women from 3 European populations Am J Clin Nutr 2009 89 354 362 19056552 10.3945/ajcn.2008.26661

[B15] de Lorgeril M Salen P Martin JL Boucher F de Leiris J Interactions of wine drinking with omega-3 fatty acids in patients with coronary heart disease: a fish-like effect of moderate wine drinking Am Heart J 2008 155 175 181 10.1016/j.ahj.2007.08.009 18082510

[B16] Hui C Qi X Qianyong Z Xiaoli P Jundong Z Mantian M Flavonoids, flavonoid subclasses and breast cancer risk: a meta-analysis of epidemiologic studies PLoS One 2013 8 e54318 10.1371/journal.pone.0054318 23349849 PMC3548848

[B17] van Duynhoven J Vaughan EE Jacobs DM Kemperman RA van Velzen EJ Gross G Roger LC Possemiers S Smilde AK Doré J Westerhuis JA Van de Wiele T Metabolic fate of polyphenols in the human superorganism Proc Natl Acad Sci U S A 2011 108 4531 4538 10.1073/pnas.1000098107 20615997 PMC3063601

[B18] Dong JY Qin LQ Soy isoflavones consumption and risk of breast cancer incidence or recurrence: a meta-analysis of prospective studies Breast Cancer Res Treat 2011 125 315 323 10.1007/s10549-010-1270-8 21113655

[B19] Setchell KD Brown NM Summer S King EC Heubi JE Cole S Guy T Hokin B Dietary factors influence production of the soy isoflavone metabolite s-(−)equol in healthy adults J Nutr 1950–1958 2013 143 10.3945/jn.113.179564 PMC3827640 24089421

[B20] Minatoya M Kutomi G Asakura S Otokozawa S Sugiyama Y Nagata Y Mori M Hirata K Equol, adiponectin, insulin levels and risk of breast cancer Asian Pac J Cancer Prev 2013 14 2191 2199 10.7314/APJCP.2013.14.4.2191 23725111

[B21] Touillaud MS Thiébaut AC Fournier A Niravong M Boutron-Ruault MC Clavel-Chapelon F Dietary lignan intake and postmenopausal breast cancer risk by estrogen and progesterone receptor status J Natl Cancer Inst 2007 99 475 486 10.1093/jnci/djk096 17374837 PMC2292813

[B22] Zaineddin AK Vrieling A Buck K Becker S Linseisen J Flesch-Janys D Kaaks R Chang-Claude J Serum enterolactone and postmenopausal breast cancer risk by estrogen, progesterone and herceptin 2 receptor status Int J Cancer 2012 130 1401 1410 10.1002/ijc.26157 21544804

[B23] Dangour AD Dodhia SK Hayter A Hayter A Aikenhead A Allen E Lock C Uauy R Comparison of composition (nutrients and other substances) of organically and conventionally produced foodstuffs: a systematic review of the available literature. Report for the Food Standards Age ncy, July 2009, contract number: PAU221 Available from: http://webarchive.nationalarchives.gov.uk/20120206100416/http://food.gov.uk/multimedia/pdfs/organicreviewappendices.pdf (accessed 14 January 2014)

[B24] Benbrook C Davis DR Andrews PK Methodologic flaws in selecting studies and comparing nutrient concentrations led Dangour et al. to miss the emerging forest amid the trees Am J Clin Nutr 2009 90 1700 1701 author reply 1701 10.3945/ajcn.2009.28605 19846547

[B25] Benbrook C Zhao X Yanez J Davies N Andrews P New evidence confirms the nutritional superiority of plant-based organic foods. An Organic Center State of Science Review 2008 Available from: http://www.organic-center.org/science.nutri.php?action=view&report_id=126 (accessed 28 January 2014)

[B26] Hallmann E Lipowski J Marszałek K Rembiałkowska E The seasonal variation in bioactive compounds content in juice from organic and non-organic tomatoes Plant Foods Hum Nutr 2013 68 171 176 10.1007/s11130-013-0352-2 23609833 PMC3659276

[B27] Benbrook CM Butler G Latif MA Leifert C Davis DR Organic production enhances milk nutritional quality by shifting fatty acid composition: a United States-wide, 18-month study PLoS One 2013 8 e82429 10.1371/journal.pone.0082429 24349282 PMC3857247

[B28] Ellis KA Innocent G Grove-White D Cripps P McLean WG Howard CV Mihm M Comparing the fatty acid composition of organic and conventional milk J Dairy Sci 1938–1950 2006 89 10.3168/jds.S0022-0302(06)72261-5 16702257

[B29] Tsiplakou E Kotrotsios V Hadjigeorgiou I Zervas G Differences in sheep and goats milk fatty acid profile between conventional and organic farming systems J Dairy Res 2010 77 343 349 10.1017/S0022029910000270 20482951

[B30] Reynaud A Fraisse D Cornu A Farruggia A Pujos-Guillot E Besle JM Martin B Lamaison JL Paquet D Doreau M Graulet B Variation in content and composition of phenolic compounds in permanent pastures according to botanical variation J Agric Food Chem 2010 58 5485 5494 10.1021/jf1000293 20394420

[B31] McAfee AJ McSorley EM Cuskelly GJ Fearon AM Moss BW Beattie JA Wallace JM Bonham MP Strain JJ Red meat from animals offered a grass diet increases plasma and platelet n-3 PUFA in healthy consumers Br J Nutr 2011 105 80 89 10.1017/S0007114510003090 20807460

[B32] Recio-Vega R Velazco-Rodriguez V Ocampo-Gómez G Hernandez-Gonzalez S Ruiz-Flores P Lopez-Marquez F Serum levels of polychlorinated biphenyls in Mexican women and breast cancer risk J Appl Toxicol 2011 31 270 278 10.1002/jat.1672 21480306

[B33] Brophy JT Keith MM Watterson A Park R Gilbertson M Maticka-Tyndale E Beck M Abu-Zahra H Schneider K Reinhartz A Dematteo R Luginaah I Breast cancer risk in relation to occupations with exposure to carcinogens and endocrine disruptors: a Canadian case–control study Environ Health 2012 11 87 10.1186/1476-069X-11-87 23164221 PMC3533941

[B34] Stahlhut RW van Wijngaarden E Dye TD Cook S Swan SH Concentrations of urinary phthalate metabolites are associated with increased waist circumference and insulin resistance in adult U.S. males Environ Health Perspect 2007 115 876 882 10.1289/ehp.9882 17589594 PMC1892109

[B35] de Lorgeril M Salen P Defaye P Rabaeus M Recent findings on the health effects of omega-3 fatty acids and statins, and their interactions: do statins inhibit omega-3? BMC Med 2013 11 5 10.1186/1741-7015-11-5 23289647 PMC3571733

[B36] Culver AL Ockene IS Balasubramanian R Olendzki BC Sepavich DM Wactawski-Wende J Manson JE Qiao Y Liu S Merriam PA Rahilly-Tierny C Thomas F Berger JS Ockene JK Curb JD Ma Y Statin use and risk of diabetes mellitus in postmenopausal women in the Women's Health Initiative Arch Intern Med 2012 172 144 152 10.1001/archinternmed.2011.625 22231607

[B37] Larsen S Stride N Hey-Mogensen M Hansen CN Bang LE Bundgaard H Nielsen LB Helge JW Dela F Simvastatin effects on skeletal muscle: relation to decreased mitochondrial function and glucose intolerance J Am Coll Cardiol 2013 61 44 53 23287371 10.1016/j.jacc.2012.09.036

[B38] Wallace DC Mitochondria and cancer Nat Rev Cancer 2012 12 685 698 10.1038/nrc3365 23001348 PMC4371788

[B39] McTiernan A Kooperberg C White E Wilcox S Coates R Adams-Campbell LL Woods N Ockene J Women's Health Initiative Cohort Study Women's Health Initiative Cohort Study. Recreational physical activity and the risk of breast cancer in postmenopausal women: the Women's Health Initiative Cohort Study JAMA 2003 290 1331 1336 10.1001/jama.290.10.1331 12966124

[B40] de Lorgeril M Mediterranean diet and cardiovascular disease: historical perspective and latest evidence Curr Atheroscler Rep 2013 15 370 24105622 10.1007/s11883-013-0370-4

[B41] Salen P de Lorgeril M The Okinawan diet: a modern view of an ancestral healthy lifestyle World Rev Nutr Diet 2011 102 114 123 21865825 10.1159/000327799

[B42] Kushi LH Doyle C McCullough M Rock CL Demark-Wahnefried W Bandera EV Gapstur S Patel AV Andrews K Gansler T American Cancer Society 2010 Nutrition and Physical Activity Guidelines Advisory Committee American Cancer Society 2010 Nutrition and Physical Activity Guidelines Advisory Committee. American Cancer Society Guidelines on nutrition and physical activity for cancer prevention: reducing the risk of cancer with healthy food choices and physical activity CA Cancer J Clin 2012 62 30 67 10.3322/caac.20140 22237782

[B43] Wolin KY Colditz GA Cancer and beyond: healthy lifestyle choices for cancer survivors J Natl Cancer Inst 2013 105 593 594 10.1093/jnci/djt052 23492347

